# Spatial Distribution and the Animal Landscape

**DOI:** 10.3201/eid1212.AC1212

**Published:** 2006-12

**Authors:** Polyxeni Potter

**Affiliations:** * Centers for Disease Control and Prevention, Atlanta, Georgia, USA

**Keywords:** Roelandt Savery, The Garden of Eden, animal painting

**Figure:**
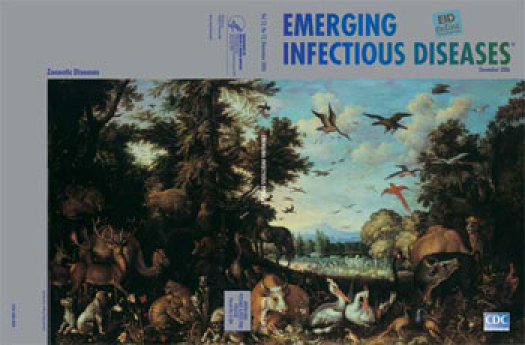
**Roelandt Savery (1576–1639).** The Garden of Eden (1618). Oil on wood (55 cm × 107 cm). National Gallery, Prague, Czech Republic

Peace and harmony reign in Roelandt Savery's The Garden of Eden, on this month's cover of Emerging Infectious Diseases. Animals sprawled across the expansive landscape move languidly, unaware of any danger to them from each other or their surroundings, their graceful contours and anatomic details of greater interest to the artist than their natural temperament. The pastoral scene lends itself to the paradoxical rendition, common in Flanders during Savery's time, of the imaginary put in realistic terms.

Flanders (in today's Belgium) was the region of Europe known for great cultural achievements, particularly in the Middle Ages and during the 16th and 17th centuries, when Flemish, also known as Netherlandish, art flourished. Landscape painting, which had been merely the setting for religious scenes, became important in its own right in the art centers of Antwerp and Brussels, and when Joachim Patinir, one of the first to create stand-alone landscapes, was praised by Albrecht Dürer as a "good landscape painter," the term landscape was elevated for the first time outside the context of Italian art (1). The genre was brought to new heights by such masters as Jan Bruegel the Elder and Peter Paul Rubens.

Contemporary of these great masters and the best-known in a family of artists, Savery was born in the Dutch village of Kortrijk and grew up in Amsterdam, where he studied under his brother Jacob and artist Hans Bol. He traveled widely and worked for Henry IV in France and Emperor Matthias in Vienna, but his stay in Prague, where he was painter and etcher of landscapes, animals, and still lifes in the court of

Rudolf II von Habsburg, produced the work for which he is most remembered (2).

Rudolf, emperor of the Holy Roman Empire and king of Bohemia and Hungary, was known for his eccentricity, one sign of which was his famed menagerie of exotic animals. Obsessive collector of unusual objects and lover of art and architecture, as well as science, the offbeat emperor surrounded himself with such artists as Bartholomeus Spranger and Giuseppe Arcimboldo, and with scientists, among them astronomers Tycho Brahe and Johannes Kepler (2). This period saw a rise in the study of mathematics, optics, physics, biology, and the development and propagation of hybrid flowers as scientific and economic pursuits.

Savery's peers in a thriving art community increasingly specialized, producing landscapes unlike any up to this time. He dabbled with and influenced several genres. His spectacular views of precipitous rocks and waterfalls influenced Dutch landscape painting. His much sought-after floral still lifes, along with those of Jan Brueghel the Elder, Ambrosius Bosschaert the Elder, and Gillis van Coninxloo, which they surpassed in tonal quality and realism, mark the beginning of the great age of Dutch flower painting (3).

The first Dutch artist to do so, Savery turned his talented hand to animal painting. Riding a strong interest in zoology at the Habsburg court and with full access to Rudolf's menagerie, he became a leader in this genre, painting and drawing a great variety of animals: pelicans, ostriches, camels, and the now extinct dodo, which he immortalized in several works. One of these, in which the dodo was the main subject, was presented to the British Museum in 1759 as painted "in Holland from the living bird" (4). Once he was paid 700 guilders, then an enormous sum, to paint "all the animals of the air and earth" (5). Soon his mythologic scenes became thinly disguised opportunities to paint more species. His favorite subjects were Orpheus charming the beasts, Noah's Ark, and scenes of Eden.

The Garden of Eden is an animal painting of the style initiated by Savery amidst increased interest in biologic research and rare creatures. The biblical narrative is only the vehicle for displaying the water buffalo, the dromedary, and all manner of unusual beasts. "Swarms of living creatures" of the mountain or the prairie, in any color, texture, shape, and form, inhabit the unlikely locale, predator frolicking with prey, desert beast with tropical. In a virtuoso display of spatial depth, Savery places Adam in the distant horizon, under the Tree of Knowledge, naming the animals "Whatsoever Adam called every living creature, that was the name thereof" (Gen. 2:19).

Naming the animals has long fascinated humans, from Aristotle to Linnaeus. And Adam's awesome task has not been completed, as indeed no one has been able to name or paint "all the animals of the air and earth." New species continue to be found (e.g., recently Mus cypriacus [6]). Others are dead as the dodo.

Like other interpretations of Eden, Savery's is not so much a geographic location as an idealized landscape. And while his beasts' exotic perfection was painted with meticulous attention to detail, the realism extended only to form, species variation clearly at odds with normal species distribution. Today, the imaginary in Savery's painting has become real. Dissimilar and diverse species from around the globe are mingled, and not just for a photo opportunity.

In collections of exotic animals, as well as in homes, parks, and the wild, far from their places of origin, animals wander the globe imported as pets, contraband cargo on board ship or plane, or in natural habitat claimed by urban and agricultural development. And contrary to notions of Eden, coexistence has been all but peaceful and harmonious. From the merger of bats, pigs, and people (Nipah virus) to the mingling of African rodents with their North American relatives (monkeypox), from AIDS to SARS to avian flu, the changing landscape of animal habitat is changing the geography and ecology of disease transmission.

## References

[1] Whinney M. Early Flemish painting. London: Faber & Faber, Ltd; 1968.

[2] Evans RJW. Rudolf II and his world: a study in the intellectual history, 1576–1612. Oxford: Clarendon Press; 1973.

[3] Bernt W. The Netherlandish painters of the seventeenth century. Oxford: Phaidon; 1948.

[4] Roelandt Savery [cited 2006 Oct 26]. Available from http://www.nhm.ac.uk/nature-online/online-ex/art-themes/caught_in_oils/more/doto_more_info.htm

[5] Roelandt Savery [cited 2006 Oct 26]. Available from http://www.figgeartmuseum.org/SiteDefault.aspx?PageID=30&nt=14&LastName=S

[6] New mouse find is "living fossil." [cited 2006 Oct 30]. Available from http://newsvote.bbc.co.uk/mpapps/pagetools/print/news.bbc.couk/1/hi/sci/tech/6043648.stm

